# Microperimetry and OCT angiography evaluation of patients with ischemic diabetic macular edema treated with monthly intravitreal bevacizumab: a pilot study

**DOI:** 10.1186/s40942-019-0176-9

**Published:** 2019-09-03

**Authors:** Felipe Pereira, Bruno Rebello Godoy, Mauricio Maia, Caio Vinicius Regatieri

**Affiliations:** 1 Departamento de Oftalmologia, Escola Paulista de Medicina, Universidade Federal de São Paulo, Rua Botucatu 821, São Paulo, SP 04023-062 Brazil; 2 New England Eye Center, Tufts Medical School, Boston, USA

**Keywords:** Diabetic macular edema, Diabetic macular ischemia, Anti-VEGF, Pharmacotherapy

## Abstract

**Background:**

Functional and anatomical evaluation of patients with ischemic diabetic macular edema after monthly injections of Bevacizumab.

**Methods:**

Five eyes from five patients with diabetic macular edema associated with macular ischemia in fluorescein angiography (FA), received 6 monthly intravitreal injections of Bevacizumab. All subjects underwent SD-OCT, FA, OCT angiography (OCTA) and microperimetry at baseline and after 6 months follow-up. Primary outcome measures were improvement of best corrected visual acuity (BCVA), microperimetry and assessment of macular perfusion (foveal avascular zone size and capillary loss).

**Results:**

Five patients completed the follow-up. BCVA improved from 20/180 to 20/74 (*p* = 0.01) and macular sensitivity improved from 11.66 to 16.26 dB (*p* < 0.007). We also observed that areas of ischemia on OCTA represented areas of lower macular sensitivity on microperimetry. No changes in macular perfusion status were noted.

**Conclusions:**

Monthly intravitreal Bevacizumab in patients with ischemic diabetic macular edema improved BCVA and macular sensitivity without compromise of perfusion in the macula. Capillary dropout areas in OCTA correlated with lower retinal sensitivity on microperimetry.

## Introduction

Diabetic macular ischemia (DMI) is an important category of diabetic maculopathy. Along with diabetic macular edema (DME), it is one of the major causes of vision loss in diabetic patients [1]. DMI is characterized by occlusion and loss of the macular capillary network or capillary dropout [2]. DME has been shown to be associated with blood–retina barrier breakdown and microaneurysmal leakage [3].

Microperimetry is a technique that combines eye fundus imaging with automated perimetry in a single measurement, allowing anatomical and functional correlations [4]. Studies of ischemic areas and macular non-perfusion in diabetes are sparse in the literature. Although patients with DMI demonstrate reduced macular sensitivity on microperimetry, the association with macular edema or their response to anti-VEGF treatment has not been explored [5].

To assess DMI, important data primarily concern perifoveal capillary arcade disruption, enlargement of the foveal avascular zone (FAZ) and reduction of capillary density. Since the Early Treatment Diabetic Retinopathy Study (ETDRS) Group Report 11, fluorescein angiography (FA) has been considered the gold standard for assessment of DMI [6].

Optical coherence tomography angiography (OCTA) has advantages including absence of intravenous contrast injection and the ability to distinguish superficial and deep retinal capillary plexuses [7]. Because of these advantages, research groups have reported that in the assessment of DMI, good correlations are demonstrated between FA and OCTA [2]. Nevertheless, OCTA has several limitations that need to be addressed, including segmentation and motion artifacts, especially when evaluating patients with disruption of retinal layer due to any pathology [8].

Intravitreal anti-VEGF treatment is the standard care for patients with DME; however, there are still conflicting results when we consider patients with DME associated with DMI. Some studies have reported an increased rate of capillary loss in the foveal region, theorizing that VEGF has neuroprotective effects and helps to increase volumetric blood flow [9]. However, small series of cases using several anti-VEGF antibody injections demonstrated improvement in visual acuity, although not as much as in patients without ischemia [10].

Therefore, this study aimed to evaluate the functional and anatomical effect of monthly intravitreal bevacizumab injections in patients with DME associated with DMI using FA, OCT, OCTA and microperimetry.

## Materials and methods

### Study design

This was a single-center, prospective, non-randomized, analytical-experimental study. The study was conducted at the Ophthalmology Department of the Federal University of São Paulo (UNIFESP), and it was approved by the Research Ethics Committee of UNIFESP under Protocol No. 71643617.0.0000.5505 and carried out in accordance with the tenets of the Declaration of Helsinki. Written informed consent was obtained from all patients before their participation in the study.

Inclusion criteria included: (1) clinically significant macular edema according to ETDRS; (2) foveal avascular zone (FAZ) larger than 500 µm in diameter; (3) central macular thickness (CMT) on optical coherence tomography (OCT) greater than 250 µm; and (4) visual acuity ranging from 20/40 to 20/400.

Exclusion criteria included: (1) glycosylated hemoglobin (HbA1c) level above 10%; (2) any ocular surgery in the preceding 6 months; (3) anti-VEGF or laser treatment in the preceding 3 months; and (4) history of glaucoma or ocular hypertension.

### Baseline evaluation

At the baseline visit, a comprehensive ophthalmic evaluation was performed, including medical history, best corrected visual acuity (BCVA) testing using ETDRS charts, applanation tonometry, slit-lamp examination, dilated fundus biomicroscopy and ophthalmoscopy.

Patients underwent SD-OCT (Spectralis HRA-OCT; Heidelberg Engineering, Heidelberg, Germany) using a 6 × 6 mm volume scanning protocol. CMT was determined using the built-in software and defined as the average thickness of a central macular area of 1000 μm in diameter centered on the patient’s foveola.

FA was performed using HRA (Heidelberg Engineering, Heidelberg, Germany). To better assess FAZ and other ischemic changes, a high-quality image centered on the fovea was obtained between 20 and 40 s after contrast injection. Macular ischemia was dual-graded by two masked assessors using protocols and standard photographs from ETDRS Report No. 11 [6]. Depending on these criteria, DMI was classified using the capillary loss parameter as none, questionable, mild, moderate or severe. FAZ was measured manually using the built-in software.

For OCTA (Triton; Topcon, Japan) analysis, we used a 3 × 3 mm or 4.5 × 4.5 mm scan centered on the fovea. FAZ measurement was done manually using Triton software. Ischemia was assessed using the same ETDRS criteria as for FA images.

Microperimetry (MAIA; Centervue, Padova, Italy) was used to quantify macular sensitivity. Pre-test training was performed with each subject. The strategy used was 37 stimuli inside a 10-degree field of vision centered on the fixation of the patient. We evaluated the average macular sensitivity threshold and fixation stability. All subjects underwent microperimetry with dilated pupils.

### Treatment and follow-up

All patients received monthly intravitreal injections of bevacizumab (Avastin; Genentech Inc, San Francisco, CA, USA) for 6 months. Patients were scheduled for follow-up examination at baseline, and at 3 and 6 months after treatment. Determination of BCVA and intraocular pressure and OCT were done every month. OCTA, microperimetry and FA were performed at baseline and after 6 months of treatment.

### Statistical analysis

Data are expressed as mean ± standard error of the mean. Statistical analyses were performed using one-way analysis of variance followed by the Tukey multiple comparison post-test. Pearson correlation coefficients were used to evaluate the correlations between the macular sensitivity,BCVA, FAZ area on OCTA and FA. A 95% confidence interval and a 5% level of significance were adopted; therefore, the results with *p* ≦ 0.05 were considered significant. All statistics were calculated using GraphPad Prism 5.0 software for Windows.

### Availability of materials and data disclosure

There is no material or data disclosure.

## Results

Five patients completed the 6-month follow-up. All patients were male and the mean age was 62 years, range 47–75 years. The mean HbA1c level was 8.0. All subjects had undergone complete panretinal photocoagulation prior to the study due to proliferative diabetic retinopathy. Three patients had had anti-VEGF injections prior to initiation of the study, and two patients were naive to anti-VEGF treatment.

Mean BCVA was 20/180 at baseline, varying from 20/100 to 20/400. After 6 intravitreal injections of Bevacizumab, four patients had BCVA improvement and one remained stable. Mean BCVA after 6 monthly injections was 20/74 (range 20/40–20/100) (*p* = 0.01).

**Fig. 1:**
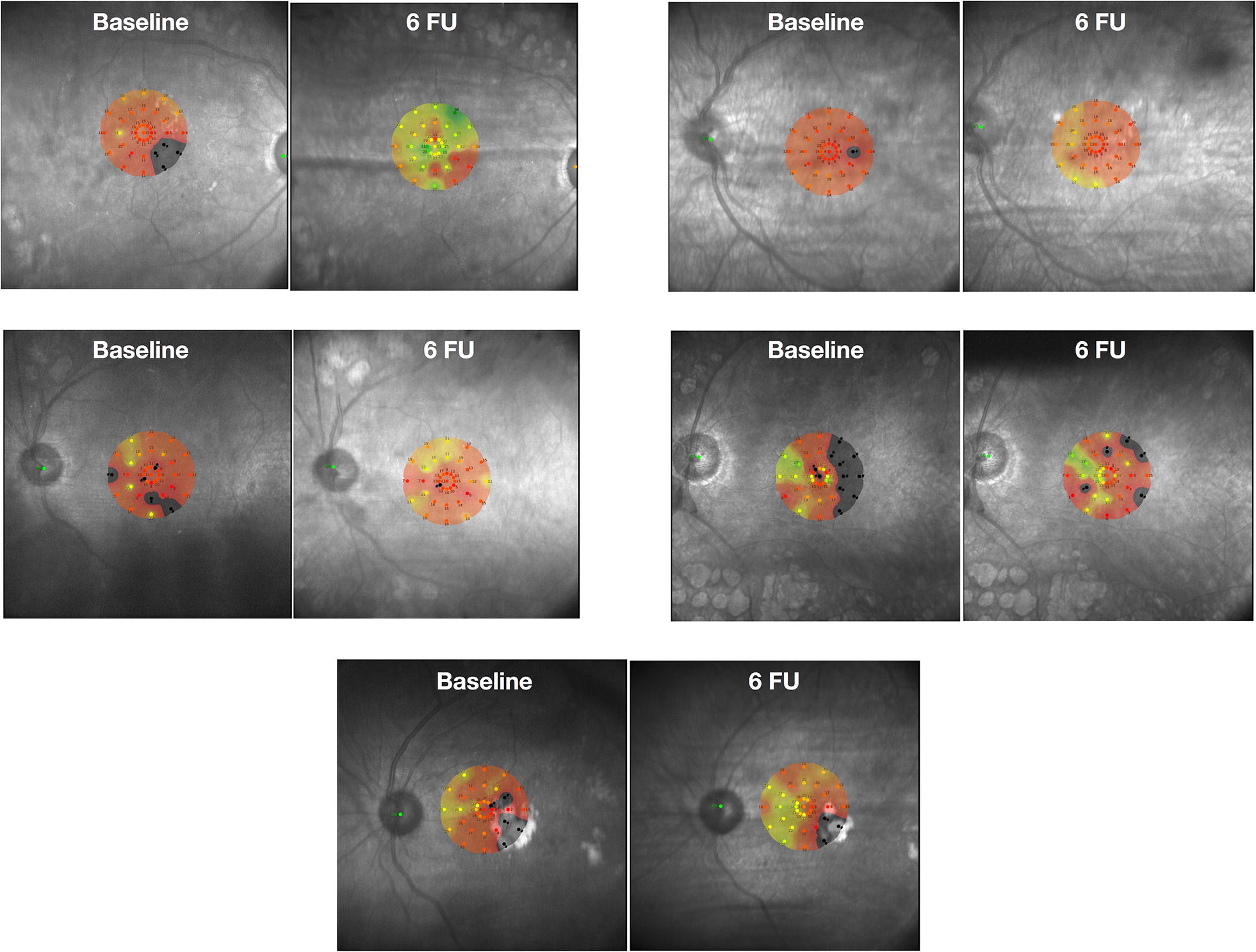
Microperimetry sensitivity of all patients at baseline and after 6 months follow-up. FU: follow-up

Fixation was considered stable in one subject, relatively unstable in three subjects and unstable in one subject. After final follow-up, this measurement was at least the same or better: two with stable fixation and three with relatively unstable fixation. Retinal sensitivity, measured by microperimetry, improved in all patients (Fig. 1). Threshold at baseline was 11.66 ± 0.77 dB, ranging from 10.7 to 12.8 dB, and it improved to 16.26 ± 3.29 dB, ranging between 13.3 and 21.8 dB (*p* = 0.007). Microperimetry improvement was more correlated with retinal thickness reduction than with visual acuity or ischemic areas on FA or OCTA.

**Fig. 2:**
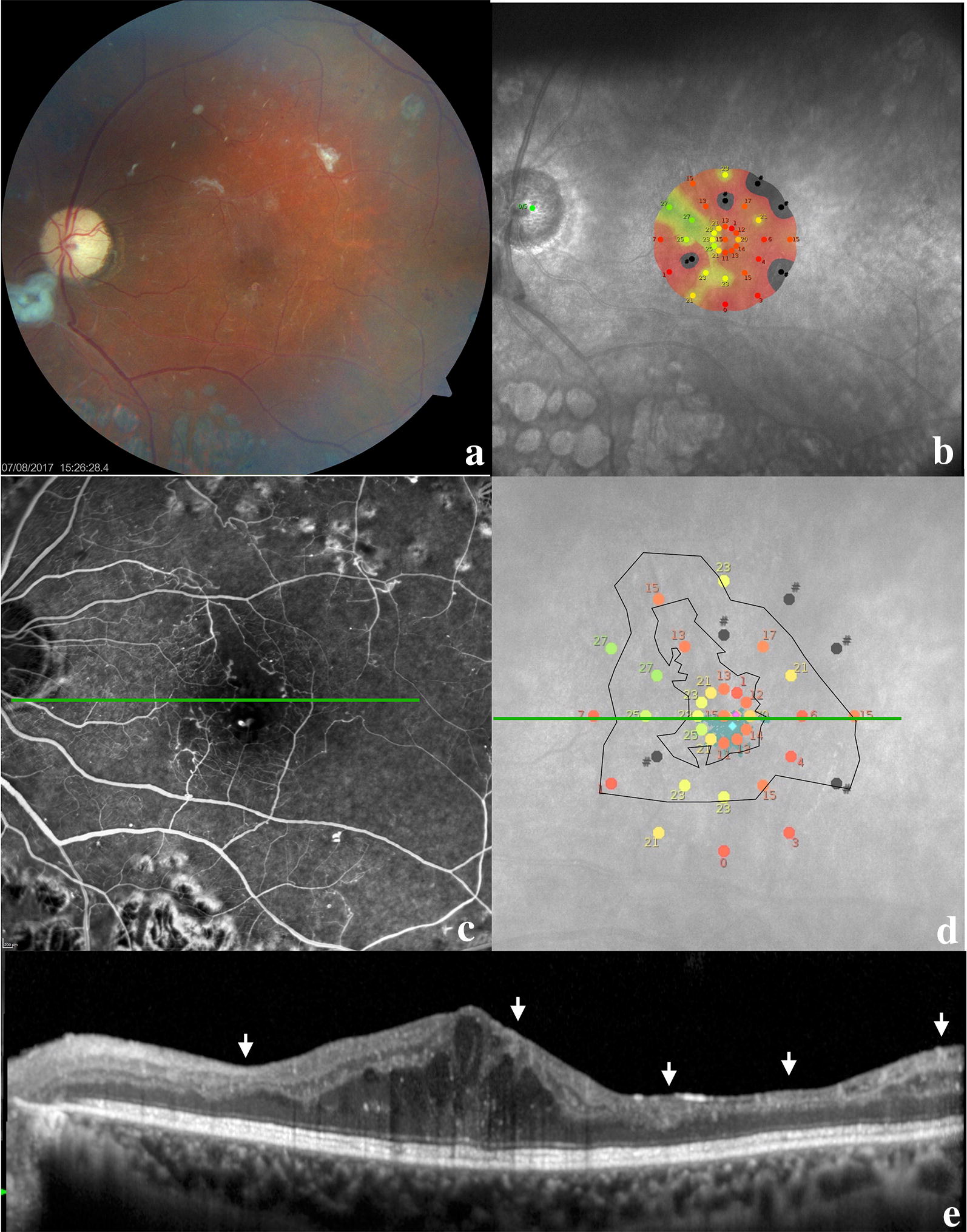
Multimodal image of a patient at 6 months follow-up. **a** Color retinography with pallor of the optic disc, macula with edema and fibrotic tissue, small hemorrhagic dots in the posterior pole, and panretinal photocoagulation beyond the temporal arcades; **b** microperimetry demonstrating macular sensitivity; **c** fluorescein angiography in the venous phase demonstrating a large foveal avascular zone (FAZ) with capillary dropout at the border of the FAZ; **d** macular sensitivity superimposed on the FAZ area (smaller black circle) and the area with some degree of hypoperfusion (larger black circle); **e** this OCT line represents the green line in the middle images; the image demonstrates the persistence of edema and the disorganization of retinal inner layers (DRIL) as indicated by the white arrows; these locations correlate with worse macular sensitivity on microperimetry and capillary dropout and hypoperfusion on fluorescein angiography

Four patients demonstrated a decrease in foveal thickness, which was more than 10% in three. Only one patient remained stable during follow-up; nevertheless, his BCVA improved from 20/100 to 20/50. Mean foveal thickness decreased from 477.8 μm (range 321–722 μm) to 341.4 μm (range 215–453 μm) after treatment (*p* = 0.008). All patients demonstrated Disorganization of the Retinal Inner Layers (DRIL) and this correlated with non-perfusion areas on FA/OCTA and with decreases in retinal sensitivity on microperimetry (Fig. 2).

All patients underwent FA at baseline and after 6 months of treatment. Mean FAZ area on FA at baseline was 1.35 ± 1.44 mm^2^ and ranged 0.22–3.77 mm^2^. At 6 months follow-up, mean FAZ area was 1.02 ± 1.02 mm^2^ and ranged 0.12–2.08 mm^2^ (*p* = 0.19). Using the ETDRS criteria for DMI, three patients were classified as severe and two as moderate. After the end of the follow-up period, no patient had a change in DMI classification. Assessing each patient, we found three with an increase in FAZ area (two with severe DMI and one with moderate DMI).

**Fig. 3:**
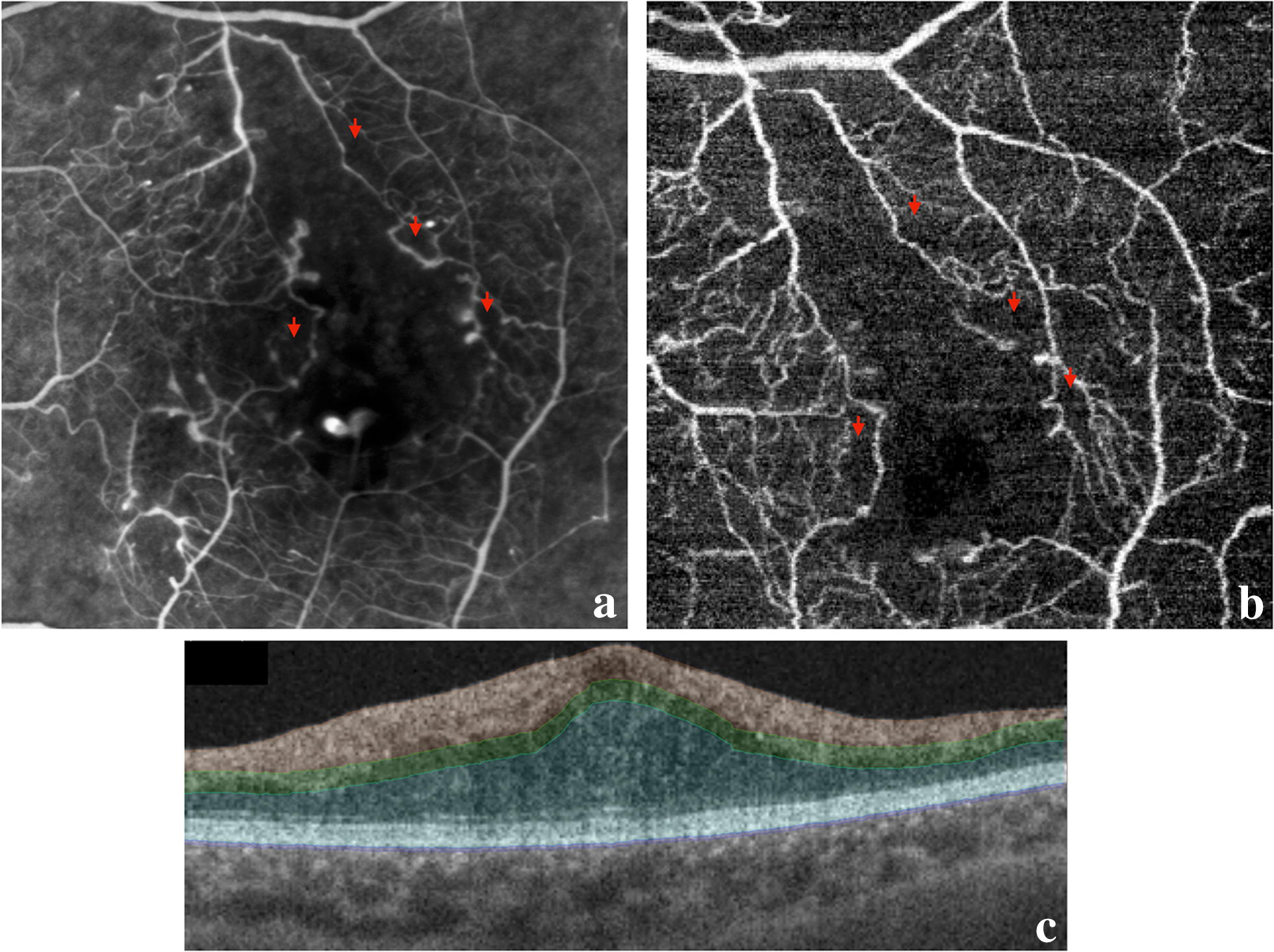
Comparison between fluorescein angiography and OCTA. **a** Fluorescein angiography, **b** OCTA image. The red arrows are placed at correlated points and represent areas with capillary dropout around the FAZ border. Note the correspondence between the two examinations; **c** segmentation lines from the OCTA examination

OCTA was performed in four patients, and in one of which, FAZ area could not be measured because of dense hard exudate with artifact projection in the examination. FAZ area measured in the three patients was 0.82 ± 0.55 mm^2^ (0.23–1.33 mm^2^) at baseline and 0.92 ± 0.57 mm^2^ (0.25–1.42 mm^2^) at 6 months follow-up, and this change was statistically significant (p = 0.02). When assessing DMI classification by OCTA using the same ETDRS criteria, the results matched those with FA (Fig. 3). The anatomical and functional results of all patients are summarized in Table 1.

**Table 1:** Anatomical and functional measurement of all subjects

	Patient 1	Patient 2	Patient 3	Patient 4	Patient 5
BCVA baseline	20/400	20/200	20/100	20/100	20/100
BCVA 6 months	20/100	20/80	20/100	20/50	20/40
Macular sensitivity baseline (dB)	11.3	11.7	12.8	10.7	11.8
Macular sensitivity 6 months (dB)	15.2	21.8	16.4	14.6	13.3
OCT foveal thickness baseline (um)	474	722	423	321	449
OCT foveal thickness 6 months (um)	371	275	393	215	453
FAZ measure—FA baseline (mm^2^)	0.22	3.77	0.32	0.98	1.47
FAZ measure—FA 6 months (mm^2^)	0.12	2.08	0.41	0.99	1.54
FAZ measure—OCTA baseline (mm^2^)	0.23	NA	NA	0.89	1.33
FAZ measure—OCTA 6 months (mm^2^)	0.25	NA	NA	1.03	1.42

*BCVA* best correct visaul acuity; *OCT* optical coherence tomography; *FAZ* foveal avascular zone; *FA*: fluorescein angiography; *OCTA* optical coherence tomography with angiography

## Discussion

Our data suggest that patients with DME associated with moderate or severe macular ischemia can show improvement in BCVA and macular sensitivity after 6 monthly intravitreal injections of Bevacizumab. Due to a lack of randomized clinical trials for anti-VEGF agents in patients with DME associated with DMI, there is a concern that decreases in VEGF levels could lead to a decrease in capillary density as well as FAZ increase with consequent loss of vision. Animal models suggest that anti-VEGF treatment is associated with retinal capillary loss [11, 12]. Furthermore, numerous case reports have correlated increased retinal non-perfusion with anti-VEGF injections in Retinal Vein Occlusion (RVO) and DME [13–15]. Nevertheless, Campochiaro et al. showed that elevated VEGF levels were associated with capillary non-perfusion, and eyes treated with anti-VEGF antibodies for RVO/DME had reduced rates of development of capillary non-perfusion [16, 17]. The BOLT study is the only prospective, randomized trial in which a quantitative analysis of macular perfusion status was provided before and after anti-VEGF treatment. No statistically significant worsening of macular perfusion status was evident, but patients with severe capillary loss according to ETDRS were not included in that study [18].

Macular sensitivity is an important predictor of visual function. Visual acuity is just one aspect of macular function, although numerous studies have presented visual acuity as the only functional parameter outcome. To our knowledge, the present report was the first to evaluate the efficacy of anti-VEGF treatment in patients with DME and DMI using microperimetry, SD-OCT and OCTA. Our findings suggest that treatment with anti-VEGF antibodies can improve light sensitivity and can provide better fixation stability to patients with DME and DMI. Notably, all patients achieved visual improvement at the final visit, which was associated with significant improvement in retinal sensitivity, but this was not necessarily accompanied by significant visual acuity improvement. Moreover, areas of lower sensitivity matched areas of capillary dropout on FA and OCTA. However, the improvement in mean threshold was not correlated with ischemia severity; rather it correlated with the reduction of retinal thickness on SD-OCT. Other authors have suggested that microperimetry can be used to evaluate visual outcome after intervention in eyes affected by DME and that this modality offers the possibility of a direct comparison of retinal pathology with psychophysical measurements as well as an objective evaluation of fixation patterns [19]. Cennamo et al. demonstrated that patients with DMI and had a significant reduction in light sensitivity compared with the control group. They also observed a correlation between areas of lower light sensitivity and structural damage on SD-OCT, particularly in the ganglion cell complex (GCC) [5].

SD-OCT analysis demonstrated a mean reduction in subfoveal retina thickness of 136.4 μm at 6 months follow-up. Four out of five patients had a reduction greater than 10%. Only one remained stable (449 μm at baseline and 453 μm at the end of follow-up).

All subjects presented with DRIL to some degree. The extent of DRIL was not affected by the treatment, and there was no correlation with final visual acuity. This could be due to our small sample size, as the literature reported good correlation between visual acuity and DRIL extent, with a stronger predictive value than even retinal thickness [20]. However, we found a correlation between DRIL area, capillary dropout on FA/OCTA and diminished retinal sensitivity on microperimetry. Moen et al. also demonstrated a correlation between alterations in inner retinal layer with ischemic areas. They theorized that superficial and deep capillary plexuses of the retina have a role as a framework for retinal cells; once this framework is lost, function (macular sensitivity) could be compromised [21].

Several authors have studied the correlation between FA and OCTA and most of them were able to correlate both examinations with respect to FAZ parameters [22, 23]. Our study corroborated these findings since all patients were classified with the same macular ischemia grade using the two examinations. However, FAZ area measurements were different between FA and OCTA, which can be explained by the differences in the subjects studied (three patients with OCTA and five patients with FA). When analyzing only patients who underwent both examinations, the results were similar.

## Conclusion

In view of the small sample size in our study, extreme caution should be taken when evaluating our results. This was a pilot study, and our goal was to demonstrate to some degree that patients with DMI graded as moderate and severe could benefit from treatment for DME with anti-VEGF antibodies, even though they did not achieve the same improvement as patients without ischemia. Microperimetry is an important functional evaluation tool, since many patients reported improvement of vision, but without compatible letter gain. We also correlated FAZ area between OCTA and FA and demonstrated that areas of internal retinal disorganization on B-scan OCT may be localized in areas with less retinal sensitivity and low perfusion on OCTA and FA.

## Data Availability

The datasets generated and/or analyzed during the current study are not publicly available due medical confidentiality but are available from the corresponding author on reasonable request.
